# Electrically and Ultrasonically Enhanced Transdermal Delivery of Methotrexate

**DOI:** 10.3390/pharmaceutics10030117

**Published:** 2018-08-05

**Authors:** Hiep X. Nguyen, Ajay K. Banga

**Affiliations:** Department of Pharmaceutical Sciences, College of Pharmacy, Mercer University, Atlanta, GA 30341, USA; Hiep.Xuan.Nguyen@live.mercer.edu

**Keywords:** iontophoresis, sonophoresis, characterization, methotrexate, transdermal delivery

## Abstract

In this study, we used sonophoresis and iontophoresis to enhance the in vitro delivery of methotrexate through human cadaver skin. Iontophoresis was applied for 60 min at a 0.4 mA/sq·cm current density, while low-frequency sonophoresis was applied at a 20 kHz frequency (2 min application, and 6.9 W/sq·cm intensity). The treated skin was characterized by dye binding, transepidermal water loss, skin electrical resistance, and skin temperature measurement. Both sonophoresis and iontophoresis resulted in a significant reduction in skin electrical resistance as well as a marked increase in transepidermal water loss value (*p* < 0.05). Furthermore, the ultrasonic waves resulted in a significant increase in skin temperature (*p* < 0.05). In permeation studies, the use of iontophoresis led to a significantly higher drug permeability than the untreated group (*n* = 4, *p* < 0.05). The skin became markedly more permeable to methotrexate after the treatment by sonophoresis than by iontophoresis (*p* < 0.01). A synergistic effect for the combined application of sonophoresis and iontophoresis was also observed. Drug distribution in the skin layers revealed a significantly higher level of methotrexate in the sonicated skin than that in iontophoresis and untreated groups. Iontophoresis and low-frequency sonophoresis were found to enhance the transdermal and intradermal delivery of methotrexate in vitro.

## 1. Introduction

Transdermal drug delivery offers several advantages, such as patient compliance, enhanced bioavailability, and avoidance of first-pass hepatic metabolism [1,2]. However, these advantages can be achieved only if therapeutically desired blood levels of drugs are obtained. Topical or transdermal drug delivery is limited by the skin barrier function—the uppermost lipophilic layer of stratum corneum, which is selectively permeable to certain chemicals [3,4]. In general, passive penetration is constrained to small molecules (molecular weight <500 Da) that are potent, with a low therapeutic dose, and have moderate lipophilicity (log P = 1–3) [5,6,7]. Drug delivery through the skin could be influenced by several factors, including skin integrity, the properties of the compound, and the composition of the formulation [8]. Transdermal drug delivery could be enhanced by optimizing the drug formulations or disrupting the integrity of the skin barrier using chemical penetration enhancers or physical methods, such as microneedles, laser, sonophoresis, and iontophoresis (ITP) [9,10,11,12].

Sonophoresis implies the application of ultrasound energy to disrupt the skin barrier to drive various therapeutic agents into and across the skin [13,14]. Sonophoresis has been operated at a frequency range from 20 kHz to 16 MHz and ultrasound intensities up to 14 W/sq·cm to enhance skin permeability [15,16]. Sonophoresis could be operated at low frequencies (20–100 kHz) or therapeutic frequencies (1–3 MHz) [9]. The mechanism of sonophoresis-enhanced drug delivery is yet to be understood [4,9,17]. Several mechanisms have been proposed, such as the thermal effects generated by the skin absorption of ultrasound energy, and cavitation effects by the collapse and oscillation of cavitation bubbles during the ultrasound application. Acoustic cavitation (formation and oscillation of gas microbubbles in the ultrasound field to disrupt the lipid bilayers of the stratum corneum) has been postulated as the predominant mechanism responsible for the sonophoresis-mediated enhancement of transdermal drug delivery [18,19,20]. Numerous studies have shown the effectiveness of sonophoresis (especially low-frequency sonophoresis (LFS), 20–150 kHz) to increase the skin permeability to various drugs and therapeutic agents including small hydrophilic molecules and large molecular weight compounds. However, ultrasound-mediated permeation greatly varies from one drug to another [21]. The ability of sonophoresis treatment to enhance drug delivery in a safe and efficient way is of interest in clinical practice [22].

ITP (an electrical current-mediated drug delivery system) is an active energy process, which employs a low physiologically acceptable electrical current (<0.5 mA/sq·cm) to drive ionized and nonionized molecules across the skin into the body [20,23]. ITP acts based on various mechanisms including electro-osmotic (convective flow) and electrophoretic (electrorepulsion) driving forces. Transdermal transport of molecules during ITP application has been postulated to occur primarily through hair follicles and sweat glands [23]. Alvarez-Figueroa et al. have suggested iontophoretic techniques to be useful to enhance transdermal penetration of methotrexate (MTX) [24]. With a negative charge at physiological pH (pH 7.4), MTX could be delivered by electrorepulsion in cathodal ITP. Electro-osmosis has also been found to further the delivery of negatively charged molecules. Tiwari et al. have performed a study on the electrically assisted delivery of MTX and indicated that a short period of the current application was therapeutically efficient to treat recalcitrant psoriasis [25].

MTX is a folic acid antagonist with the antineoplastic activity that has been employed to treat psoriasis and rheumatoid arthritis [26]. Mechanistically, MTX competitively inhibits the enzyme dihydrofolate reductase, thus inhibiting mitotic activity and DNA synthesis [27]. In general, MTX is administered by either parenteral or oral route for the treatment of psoriasis and rheumatoid arthritis [24,26]. Prolonged exposure to MTX systemically may be associated with several side effects, such as hepatotoxicity, suppression of bone marrow function, dyspnea, leukopenia, anemia, and thrombocytopenia [28]. MTX delivery into skin helps to reduce systemic exposure and bypass hepatic metabolism [26]. However, transdermal delivery of MTX faces major challenges in that MTX is hydrophilic (log P = −1.85), has a moderately high molecular weight (454.44 Da), and is mostly in dissociated form at physiological pH; thus, passive permeation across the skin is limited. MTX has been formulated in various formulations, and the delivery has been reported to be increased using enhancement methods [24,26,29,30]. In this study, we aimed to employ cathodal ITP, anodal ITP, and LFS to enhance the delivery of MTX into and across dermatomed human cadaver skin in vitro.

## 2. Materials and Methods

### 2.1. Materials

MTX was purchased from Sigma Aldrich (St. Louis, MO, USA). Phosphate buffered saline (PBS) (0.1 M, pH 7.4 ± 0.1) was obtained from Fisher Scientific (Fisher BioReagent, Springfield, NJ, USA). Methylene blue was purchased from Eastman Kodak Co. (Rochester, NY, USA), D-Squame stripping discs from CuDerm (Dallas, TX, USA), while cotton swabs were obtained from Dynarex (Orangeburg, NY, USA). Silver chloride electrodes and silver wire were procured from ALA Scientific Instruments (Farmingdale, NY, USA) and Alfa Aesar (Ward Hill, MA, USA), respectively. Human cadaver skin tissues (0.26 ± 0.04 mm thick, *n* = 24) were obtained from the New York Fire Fighter skin bank (Presbyterian Hospital, New York, NY, USA).

### 2.2. Experimental Conditions and Apparatus

**Figure 1:**
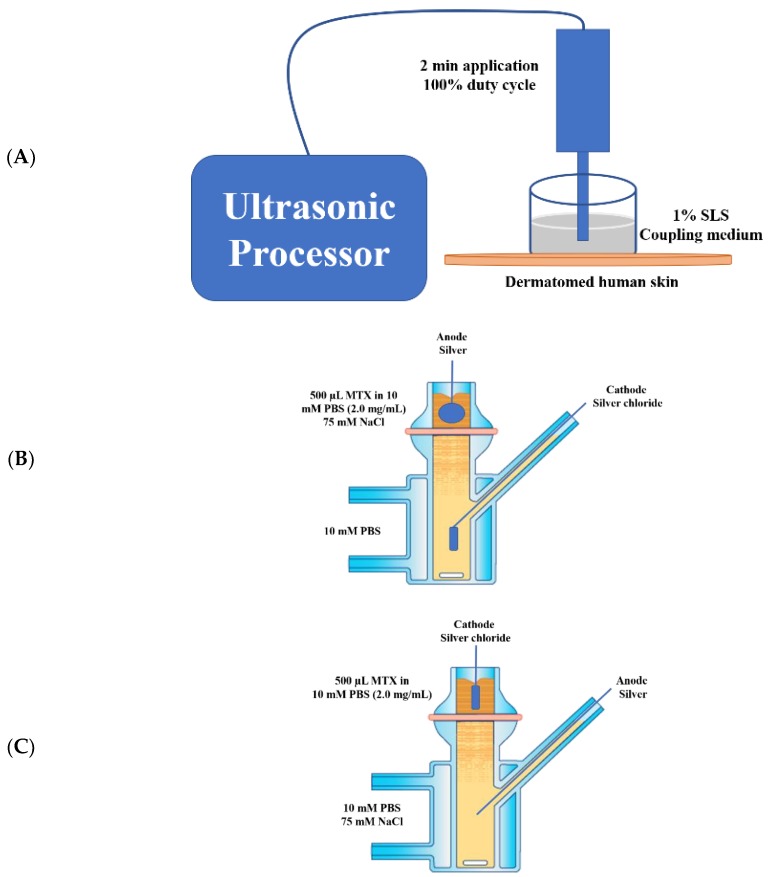
Experimental setup of (**A**) low-frequency sonophoresis (LFS), (**B**) anodal iontophoresis (anodal ITP), and (**C**) cathodal iontophoresis (cathodal ITP).

LFS has been used to enhance transdermal delivery of various molecules [9]. Sonophoresis was carried out using a low-frequency sonicator at 20 kHz frequency and 6.9 W/sq·cm ultrasound intensity (VCX 500, Sonics and Materials, Newtown, CT, USA) for 2 min application, amplitude of 30%, and duty cycle of 100%. The distance from the horn to the skin surface was 300 µm while 1% (*w*/*v*) sodium lauryl sulfate (SLS, surfactant solution) was used as the coupling medium to transmit the ultrasound energy to the skin (Figure 1A) [4,9,31]. A piece of dermatomed cadaver human skin was mounted on a layer of parafilm and under the sonicator horn in which the stratum corneum was facing upwards, towards, and perpendicular to the horn. A wider donor top—cylindrically shaped chamber, made of glass (17.87 mm diameter), open from both ends to contain the sonicator probe—was then placed over the skin [9]. The sonicator horn was dipped in the SLS medium (1.5 mL) and the ultrasound was generated for 2 min. The sonication system was “tuned” prior to each experiment, in accord with a procedure provided by the manufacturer [4]. After the sonophoretic treatment, the skin tissues were washed 3 times with 10 mM PBS, gently cleaned using Kimwipes, and then mounted on Franz vertical diffusion cells.

For ITP, the experimental setup is elaborated in Figure 1B (Anodal ITP) and Figure 1C (Cathodal ITP). Cathodal ITP was employed to drive negatively charged MTX into the skin by electrostatic repulsion [24]. The efficacy of electro-osmosis on the drug delivery was also investigated in anodal ITP. The electrodes were inserted into the donor and receptor compartments. A direct current was then applied for 60 min at 0.4 mA/sq·cm using a current source (Keithley 2400, Cleveland, OH, USA). The Franz cells were set up that the electrodes were immersed in the solution, but not touching the skin to avoid skin burn, and no air bubbles were trapped below the electrodes to avoid possible disruption in the current flow [31]. When several cells were iontophoresed, the electrodes were connected to ensure the polarity orientation [31].

### 2.3. Dye Binding Studies

We used an aqueous solution of methylene blue dye (1% *w*/*v* in DI water) to visualize the surface of dermatomed human skin after ITP or sonophoresis treatment. After the 2-min treatment by sonophoresis, or 1 h by anodal or cathodal ITP, methylene blue solution was applied on top of the skin. After 1 min, the excess dye was swabbed using Kimwipes and alcohol swabs (Curity^TM^ Covidien, Mansfield, MA, USA). Then the treated skin was visualized under a microscope (ProScopeHR Digital USB Microscope, Bodelin Technologies, Oregon City, OR, USA) [32,33].

### 2.4. Skin Integrity Measurement

After 1 h of cathodal and anodal ITP, the drug solution was gently removed using 3 sheets of Kimwipes, the skin surface was washed 3 times using 10 mM PBS before the addition of 300 µL 10 mM PBS for measuring the skin resistance. A load resistor (Silver chloride electrode, *R_L_*, 100 kΩ) was immersed in the PBS solution in the donor chamber of vertical Franz diffusion cells without touching the skin membrane (PermeGear, Hellertown, PA, USA). A silver electrode was dipped in the receptor solution (5 mL 10 mM PBS solution, pH 7.4), which was constantly kept at 37 ± 1 °C. An electrical current at a voltage of 100 mV (*V*_0_) was passed across the skin tissue (Agilent Multimeter and Agilent waveform generator, Agilent Technologies, Santa Clara, CA, USA) and the voltage drop (*V_S_*, mV) across a diffusion unit area the skin (*A*, 0.64 sq·cm) was recorded and calculated (*n* = 4) [30,32].

The skin resistance value (*R_S_*, kΩ/sq·cm) was estimated using Equation (1).

(1) $$R_{s}=\frac{R_{L}V_{s}}{A\left(V_{o}-V_{s}\right)}$$

Skin conductivity indicates the skin integrity. Particularly, a sudden decrease in the skin electrical resistance denoted the disruption of the skin barrier [11]. After skin resistance measurement, 5 sheets of Kimwipes were gently dabbed on the skin surface to remove PBS solution before the transepidermal water loss value (TEWL) was measured.

The barrier function of dermatomed human skin before and after ITP or sonophoresis were assessed by measuring TEWL (VapoMeter, Delfin Technologies Ltd., Kuopio, Finland). Treated skin samples were mounted on vertical Franz diffusion cells for the TEWL measurement (*n* = 4). The TEWL values depicted the effects of the physical treatments on the skin intactness, with an increase suggesting compromised skin [10,11]. We also measured the skin temperature before and after the physical treatments and reported the results as the increase in the skin temperature (%, *n* = 4).

### 2.5. In Vitro Permeation Studies using Vertical Franz Diffusion Cells

Permeation study has been commonly used to measure the drug transport across and into the skin [34,35]. In this study, the delivery of MTX through dermatomed human cadaver skin was investigated using vertical Franz diffusion cells (PermeGearV6, Hellertown, PA, USA). The experimental setup of Franz diffusion is listed in Table 1. The donor chamber was left open during the study, while the receptor was kept at 37 °C to maintain skin temperature at 32 °C. The receptor solution was constantly stirred at 600 rpm. Skin that was not treated by either ITP or sonophoresis was denoted as control (Passive, *n* = 4). The ultrasound-treated and untreated skin samples were clamped between donor and receptor chambers with the epidermal surface of skin facing towards the donor compartment of the cells. Drug solution (500 µL) was applied on the skin using a pipet to fully cover the skin permeation area and immerse the electrodes (Figure 1B,C). Aliquots of receptor solution (300 µL) were taken at 0, 0.5, 1, 2, 4, 6, 8, 22, and 24 h, and replaced with freshly prepared receptor fluid (300 µL). The samples were then quantitatively analyzed using the High-Performance Liquid Chromatography (HPLC) method. The cumulative amount of MTX permeated through a diffusion unit area into the receptor chamber was plotted as a function of time (permeation graph, *n* = 4). Lag time of the drug delivery was estimated as the x-intercept of the linear part (*R*^2^ > 0.95) of this curve.

The flux of MTX permeability (*J*) was estimated from the mass (*m*) of MTX passing through a cross-sectional area of the skin (*A*) during a time period (*t*), as shown in Equation (2).

(2) $$J=\frac{dm}{dtA}$$

The steady-state flux of MTX was estimated from the slope of the linear part of the permeation curve (*R^2^* > 0.95). After that, the permeability coefficient (*K_p_*, cm/h) was determined from the steady-state flux (*J*, µg/h), the concentration of MTX in the donor chamber (*C*, 2000 µg/mL), and the effective permeation area (*A*, 0.64 sq·cm), using Equation (3).

(3) $$K_{p}=\frac{J}{C\;A}$$

### 2.6. Skin Distribution Studies

The distribution of MTX in different layers of the skin was estimated after 24 h permeation studies. MTX solution remained in the donor compartment was removed using dry and receptor-wetted Q-tips, followed by tape stripping (D-Squame stripping discs D101, CuDerm, Dallas, TX, USA). The tape strips were removed quickly with forceps and contained the drug remaining on the skin surface. To measure the drug levels in the skin, the epidermis was separated from the dermis using forceps and were both minced individually using scissors. The skin pieces were then immersed in 2 mL extraction solvent (methanol: 10 mM PBS = 50:50 *v*/*v*) in 6-well plates. The plates were then constantly shaken at 100 rpm for 24 h at ambient conditions. The samples were then filtered through a 0.2 µm filter and analyzed using HPLC.

### 2.7. Quantitative Analysis

A Reversed-Phase High-Performance Liquid Chromatography (RP-HPLC) method was employed to analyze the concentration of MTX in the samples (e2695 Separating Module and photodiode array detector, Waters, Milford, MA, USA).

The mobile phase consisted of acetonitrile and potassium phosphate monobasic buffer (10 mM, pH 3.5) (13:87 *v*/*v*). A C18 Gemini-NX column (110 A, 150 × 4.6 mm^2^, 5 µm) was used at 35 °C (Phenomenex, Torrance, CA, USA). The mobile phase was passed through the column at a rate of 1.0 mL/min. The volume of sample injection was set at 10 µL while the drug was detected at 304 nm wavelength.

### 2.8. Statistical Analysis

Statistical calculations were carried out using Microsoft Excel and GraphPad Prism 5 (GraphPad Software, La Jolla, CA, USA). One-Way ANOVA and Student’s *t*-test were employed in the analysis. A statistically significant difference was depicted by a *p*-value less than 0.05 [36].

## 3. Results and Discussion

### 3.1. Dye Binding Studies

**Figure 2:**
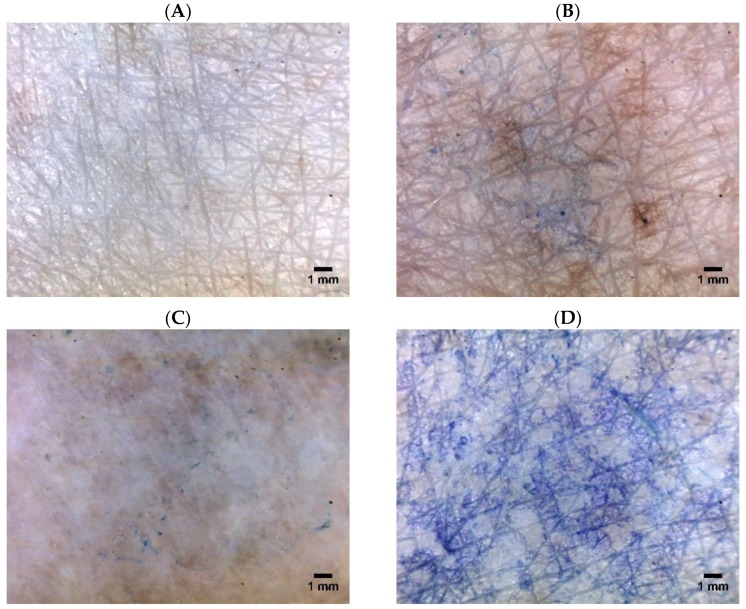
Microscopic images of (**A**) untreated dermatomed human skin and skin treated by (**B**) anodal ITP, (**C**) cathodal ITP, and (**D**) LFS.

After the physical treatments (Table 1), we applied the dye solution on the treated site of human cadaver skin. No absorption of methylene blue occurred on the untreated or intact skin (Figure 2A). This observation could be explained by the hydrophilicity of methylene blue that blocked its penetration across the stratum corneum layer of skin. At 1 h post-treatment by either anodal or cathodal ITP, the skin tissue became more accessible to the dye (Figure 2B,C). This result might be attributed to the drug transport through the skin layer during the ITP application, creating some penetration pathways, which appeared visible on the microscopic images. Furthermore, the skin gradually hydrated in contact with the donor and receptor fluids, thus increasing the permeability to the dye. The effect of ITP on the skin barrier was previously investigated using a freeze-fracture electron microscopy, light microscopy, and Fourier-transform infrared spectroscopy. The authors suggested that, at low-current densities, ITP disorganized the stratum corneum locally, whereas ITP at higher-current densities resulted in a general disruption of the stratum corneum lipid [37,38]. Furthermore, ITP was found to increase the dimensions of the hair follicles to facilitate drug penetration. Fast recovery of the skin integrity in vivo and relatively low possibility of skin irritation were expected when the current was discontinued [24].

LFS clearly disrupted the skin barrier, allowing a significant amount of dye to pass through (Figure 2D). In a previous study, we have used a confocal microscope to examine the penetration of calcein across excised hairless rat skin treated by low-frequency ultrasound [9]. Observing the histological sections of the treated skin, we found that sonophoresis enhanced the skin permeability of calcein dye. In another study, Boucaud et al. used histology and scanning electron microscopy to examine human skin treated by low-frequency ultrasound. The authors reported that when the ultrasound intensities were less than 2.5 W/sq·cm, the skin remained unchanged: a macroscopically normal skin surface and no disruption of the skin structure [4]. Differently, for hairless rat skin, 2.5 W/sq·cm ultrasound exposure led to transient and slight erythema initially; delayed and deep lesions such as dermal and muscle necrosis after 24 h. The authors did not observe any clinical changes in hairless rat skin under 1 W/sq·cm pulsed-mode ultrasound exposure, and histological sections of the treated skin were similar to those in the untreated group in vivo. However, the authors observed obvious histological skin changes including the separation of the epidermis and dermal necrosis at the ultrasound intensity of 4 W/sq·cm (continuous mode, and 10 min). The use of pulsed-mode ultrasound at 5.2 W/sq·cm resulted in significant skin modifications, such as epidermal detachment and edema of the upper dermis layer [4]. The authors reported a macroscopically second-degree burn when the ultrasound was applied in continuous mode at 7 W/sq·cm ultrasound intensity and in pulsed mode at 12.3 W/sq·cm ultrasound intensity. Thus, the inappropriate application of high-intensity sonophoresis (20 kHz) could lead to severe skin damage and lesions. In our study, we employed controlled ultrasound intensity and treatment duration to prevent skin irritation. Kost et al. used low-frequency ultrasound (5 W/sq·cm, 20 kHz, pulsed mode, and 1 h) to treat rat skin tissue and found no difference between the histological sections of untreated and sonicated skin [39]. Pires-de-Campos and colleagues employed continuous-mode sonophoresis at a 3 MHz frequency and a 0.2 W/sq·cm intensity to drive caffeine through porcine skin. Monitoring the morphological changes of the skin, the authors observed a notably thinner subcutaneous adipose tissue, damages to the adipocytes, and a decrease in the number of cells [40]. The effect of sonophoresis on skin was attributed to the cavitation and oscillation of gas pockets under the ultrasound wave [4]. The cavitation thresholds depended on the ultrasound frequency and intensity. A rapid cavitation was observed with sonophoresis operated at low frequencies and high intensities [4]. The presence of cavitation has been indicated histologically in several studies that revealed the appearance of crater-like lesions [41] or holes [42] on the hairless mice skin.

### 3.2. Skin Integrity Measurement

**Figure 3:**
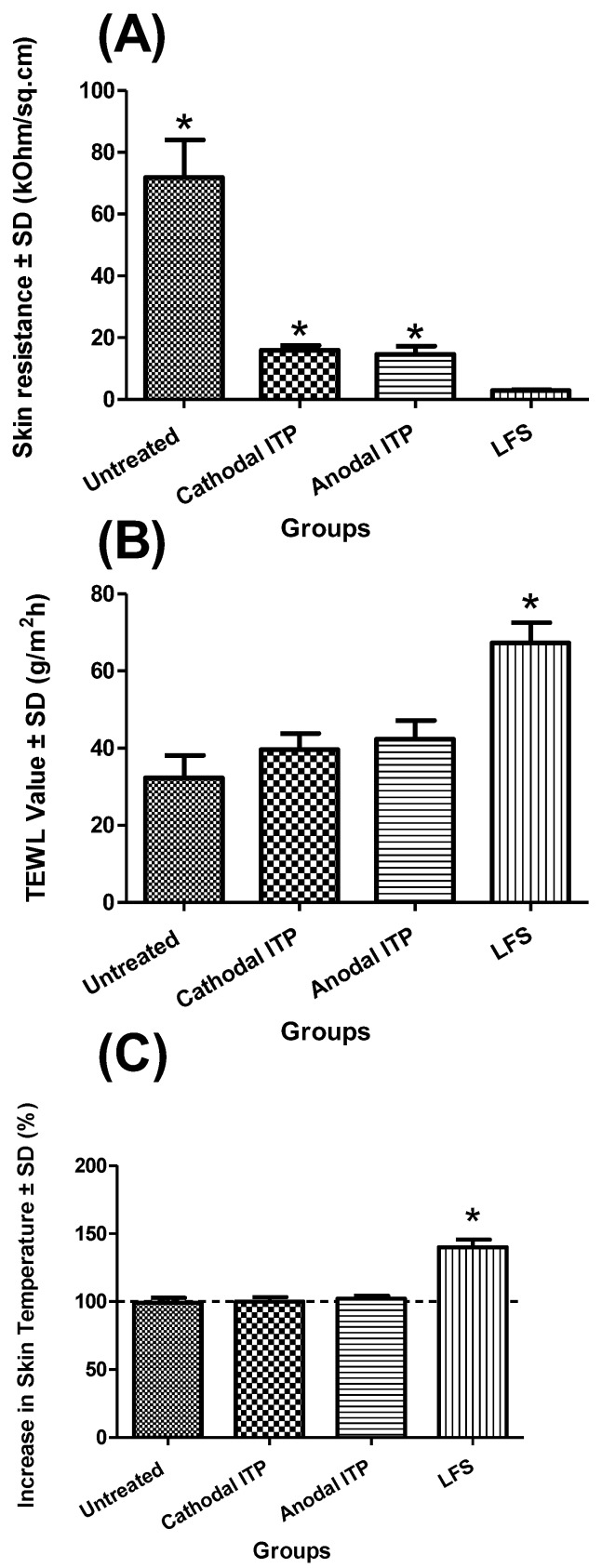
(**A**) Electrical resistance, (**B**) transepidermal water loss, and (**C**) increase in the temperature of dermatomed human cadaver skin (* indicated statistical difference from the LFS, mean ± SD, *n* = 4, *p* < 0.05).

Sonophoresis treatment led to a markedly lower skin electrical resistance value (2.90 ± 0.28 kΩ/sq·cm) than the other groups (*n* = 4, *p* < 0.05) (Figure 3). Similarly, Le et al. reported that the application of a 10-min ultrasound resulted in an increase in the electrical conductivity of cadaver pig skin by approximately 60-fold [31]. Mitragotri et al. suggested a threshold dose of ultrasound energy below which the effect of sonophoresis on skin conductivity appeared unnoticed [43]. However, when the ultrasound energy goes beyond the threshold, an increase in the applied ultrasound energy would result in an increase in both skin conductivity and skin permeability. Intact skin (passive, 71.91 ± 12.14 kΩ/sq·cm) with full skin integrity offered the highest resistance as compared to physically treated skin. No significant difference was found between anodal (14.59 ± 2.69 kΩ/sq·cm) and cathodal ITP (15.94 ± 1.63 kΩ/sq·cm) (*p* = 0.43) (Figure 3). Le et al. also measured skin conductivity and reported that the conductivity of porcine skin markedly increased following one-hour ITP and this increase lasted for more than 24 h [31]. Kalia et al. suggested that iontophoretic treatment enhanced the hydration of the stratum corneum and, thus, reduced the skin electrical resistance [44]. Tezel et al. also observed a 100-fold increase in skin conductance at the end of the 10-min application of ultrasound [45].

Changes in TEWL values could indicate possible modification of the skin integrity. A disruption of the skin barrier function would result in an enhancement of water loss through the skin tissue, thus elevating the TEWL values [46,47]. Thus, TEWL could be employed to evaluate the extent of skin barrier disruption. No marked difference was observed in TEWL values between passive and ITP groups (*p* = 0.05). LFS (67.25 ± 5.23 g/m^2^ h), resulting in a significantly higher TEWL value of cadaver human skin as compared to passive (32.30 ± 5.79 g/m^2^ h), anodal ITP (42.30 ± 4.88 g/m^2^ h), and cathodal ITP (39.63 ± 1.35 g/m^2^ h) (*p* < 0.05) (Figure 3). This observation indicated that ultrasound treatment considerably disturbed the skin structure. Further, we disclosed a negative correlation between the skin electrical resistance and TEWL values. Herwadkar et al. found that TEWL value of excised hairless rat skin increased notably from 31.6 ± 0.12 g/m^2^ h to 69.5 ± 12.6 g/m^2^ h due to the 2-mjn application of low-frequency ultrasound. However, this notable increase was absent when the ultrasound energy was applied for a shorter period (1 min) [9].

We also observed that sonophoresis significantly increased skin temperature from 99.00 ± 3.83% to 140.00 ± 5.66% (*p* < 0.05). We could attribute this result to the alteration in the skin resistance and TEWL values. Skin temperature remained unchanged after either anodal (102.00 ± 2.31%) or cathodal ITP (100.00 ± 3.27%) (*p* > 0.05). Boucaud et al. also measured the skin temperature after the sonophoresis treatment. The temperature of the dermis layer was found to be 42 °C and 39 °C following a continuous and pulsed ultrasonic exposure, respectively [4]. Furthermore, skin temperature also changed with ultrasound intensity. When the ultrasound was applied continuously for 10 min at the intensity of 7 W/sq·cm, the temperature of the skin surface reached 65 °C with the appearance of macroscopic necrosis. When the intensity was reduced to 4.5 and 2.5 W/sq·cm, skin temperature decreased accordingly to 59 °C and 42 °C. Thus, lower ultrasonic intensity could lead to less severe skin changes. Low-frequency ultrasound (20 kHz) has been shown to enhance transdermal diffusion of water molecules [48]. We could hypothesize that sonicated water molecules might diffuse from the coupling medium in the donor chamber across the skin tissue during the sonophoresis application, thus increasing skin temperature.

### 3.3. In Vitro Permeation Studies Using Vertical Franz Diffusion Cells

#### 3.3.1. Passive Permeation

Due to hydrophilicity (log P = −1.85), relatively high molecular weight, and ionization at physiological pH [28], MTX was not able to penetrate the intact skin barrier—lipophilic stratum corneum layer—in the passive group (0.00 ± 0.00 µg/sq·cm). In another study, a negligible amount of MTX was found to penetrate the skin by passive diffusion and only a sufficiently high drug concentration in the donor formulation resulted in a merely detectable MTX level in the receptor fluid [24]. Similarly, Weinstein and coworkers reported that the transdermal passive penetration of MTX from an aqueous drug solution (2% MTX) was only around 5 µg/sq·cm after 48 h [49]. Thus, the desired MTX delivery requires the use of some enhancement technologies.

#### 3.3.2. ITP-Mediated Delivery of MTX

Physical treatments (ITP and sonophoresis) significantly enhanced the drug delivery into the receptor chamber (*n* = 4, *p* < 0.05). Cathodal ITP (0.54 ± 0.07 µg/sq.cm, *p* = 0.03) as well as LFS (161.92 ± 30.06 µg/sq·cm, *p* = 0.00) markedly enhanced the drug delivery as compared to the passive group. This observation might be attributed to the negative charge of MTX at physiological pH (pK_a_ 5.6, 4.8, and 3.8) that was repelled by the negatively charged electrode (silver chloride) in the donor compartment of cathodal ITP. Previously, we have compared the in vitro delivery of MTX through cadaver hairless rat skin using either maltose microneedles or cathodal ITP, in which microneedle insertion resulted in a significant enhancement in drug delivery as compared to ITP application. Moreover, the combination of cathodal ITP with microneedles led to a comparable drug delivery as microneedles alone [26]. This result indicated a limited enhancement effect of cathodal ITP on MTX delivery. In this study, we also observed an insignificant improvement in the transdermal delivery of MTX when cathodal ITP was combined with sonophoresis as compared to sonophoresis alone. In the previous study, we reported that the passive delivery of MTX across full-thickness rat skin in vitro was negligible [30] and was enhanced approximately five-fold with the use of ITP (MTX 15 mg/mL in phosphate buffer 0.25 M, current density of 0.5 mA/sq·cm, and ITP duration of 120 min) [28]. The mechanism of ITP-induced permeability enhancement included electrorepulsion and electroosmosis [50].

**Figure 4:**
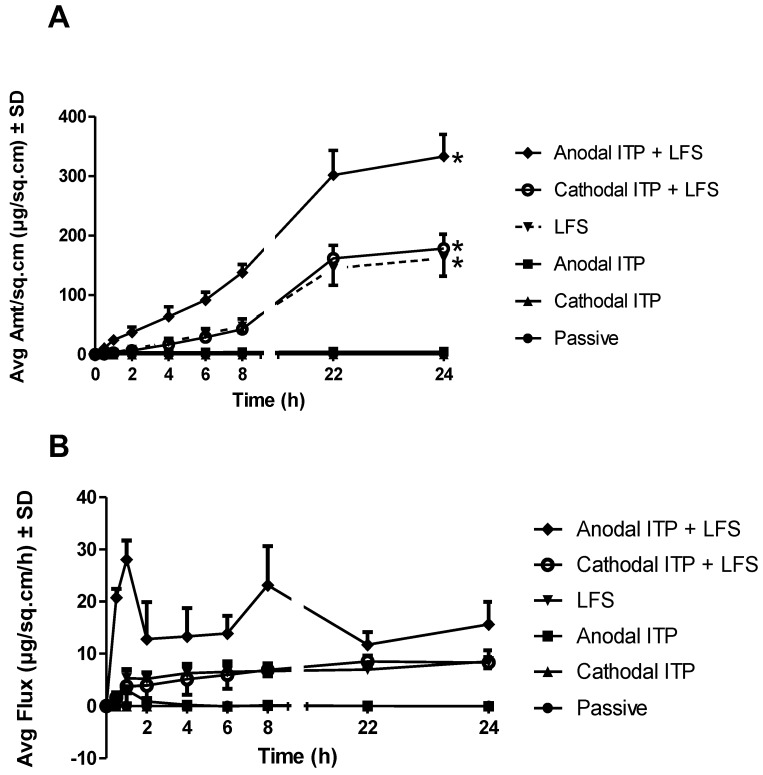
(**A**) Average cumulative amount and (**B**) flux of methotrexate (MTX) delivered through untreated, ITP, and sonophoresis-treated dermatomed human cadaver skin (* indicated statistical difference from the passive group, mean ± SD, *n* = 4, *p* < 0.05).

Anodal ITP delivered a significantly higher amount of MTX into the receptor chamber than cathodal ITP (*p* < 0.05) (Table 2, Figure 4). Anodal ITP offered an advantage of electroosmotic addition to the iontophoretic flux. This observation indicated that the electro-osmosis in anodal ITP provided a stronger driving force for MTX delivery than electrorepulsion in cathodal ITP. Interestingly, when a microdialysis technique was used to investigate the transdermal delivery of MTX through male hairless rat skin in vivo, we reported a reduction of the level of MTX in the dialysate samples after the discontinuation of ITP and removal of the drug formulation from the skin [26]. No such observation was made in our study where the drug constantly accumulated into the receptor fluid, resulting in an increasing drug amount delivered after the current was terminated while the drug solution was maintained in the donor chamber. The constant increase in the drug delivery could be more likely explained by the presence of the drug formulation on the skin surface rather than the drug depot in the skin layers since MTX, as a negatively charged, hydrophilic molecule (log P = −1.85), should permeate easily to the deeper skin layers once the stratum corneum was bypassed. The skin barrier could have been irreversibly modified after 1 h ITP in vitro, as indicated by skin electrical resistance and TEWL measurement, to facilitate further drug diffusion even after the current termination. Similarly, Le et al. employed ITP (current density of 0.45 mA/sq·cm for 1 h) to produce an immediate effect on transdermal flux of heparin and a continuous long-term enhancing effect even after the electric current was discontinued [31].

In a previous work, we have studied and optimized the iontophoretic parameters for transdermal delivery of MTX, such as phosphate buffer strength, drug concentration, the current density, and duration of application [28]. Singh and Singh reported the synergistic effect of the combination of ITP and chemical penetration enhancers on the enhanced delivery of MTX [51]. Similarly, Prasad and coworkers used modulated direct-current ITP together with ethyl acetate, ethanol, and menthol to enhance the flux of MTX by 161% [52]. Stagni and Shukla [53] and Alvarez-Figueroa et al. [24] have reported that ITP treatment markedly enhanced the skin permeation of MTX. Furthermore, the ITP-mediated delivery of MTX from hydrogel formulations was also investigated. The authors suggested that the physical iontophoretic treatment provided a more efficient drug delivery than passive diffusion [29]. Tiwari et al. reported that ITP at 1 mA/sq.cm current density could effectively treat palmer psoriasis [25]. Prasad and colleagues revealed that the use of modulated direct-current ITP led to significantly higher delivery of MTX than direct-current ITP [38]. The histopathological study was conducted on mice in vivo to show that ITP at a low current density of 0.2 mA/sq·cm was tolerated by the skin, and the ITP-induced skin disruption was recovered within 48 h [52]. Transdermal penetration of MTX was found to decline with increasing ionic strength of the drug formulation; however, it increased with increasing current density [24]. The effectiveness of MTX ITP for the treatment of psoriasis requires future studies to (i) assess the safety of iontophoretic delivery to the psoriatic skin, (ii) optimize MTX concentrations for therapeutic effect, and (iii) measure MTX iontophoretic transport and accumulation through a damage skin barrier such psoriatic skin [24].

#### 3.3.3. Sonophoresis-Mediated Delivery of MTX

LFS led to markedly enhanced cumulative delivery, permeability coefficient, and flux of MTX as compared with either anodal or cathodal ITP (*p* < 0.05) (Table 2, Figure 4). Recent literature has supported that LFS would be a prospective method to facilitate drug delivery into and across skin [9,54]. Sonophoresis-mediated skin permeability varies based on several factors including frequency, intensity, and treatment duration. LFS-mediated permeability of skin was caused by several mechanisms such as radiation pressure, cavitation, and acoustic microstreaming effects [55]. The application of ultrasound was found to produce highly permeable and localized transport regions in the skin [56]. Wolloch and Kost demonstrated that microjets offered a significantly higher contribution to the enhancement of skin permeability than shock waves [56]. Mechanistically, ultrasound pretreatment might disorder the lipid bilayers of skin and open new pathways for transdermal drug transport [31].

Pires-de-Campos and colleagues have used continuous ultrasound at 3 MHz frequency and 0.2 W/sq.cm intensity to effectively drive caffeine into and across porcine skin [40]. Previously, we employed low-frequency ultrasound (55 kHz, SonoPrep ultrasonic skin permeation device, Echo Therapeutics, Franklin, MA, USA) to deliver daniplestim across hairless rat skin in vivo and found that sonophoresis-enhanced drug delivery was dependent on the drug concentration in the formulations (2 mg/mL and 5 mg/mL). Following sonophoresis, we observed an initial increase, followed by a gradual decrease in daniplestim levels in the plasma samples [20]. Interestingly, Mitragotri et al. reported a 100-fold increase in water permeability during sonophoresis and 94% of this alteration was recovered within 2 h after the ultrasound termination [57]. Boucaud et al. reported that the ultrasound treatment (frequency 20 kHz, intensity 2.5 W/sq·cm, pulsed mode, and 10% duty cycle) led to a significantly greater water flux (2.5-fold) as compared with passive permeation [58]. Similarly, the application of low-frequency ultrasound significantly facilitated transcutaneous penetration of ketoprofen from 74.87 ± 5.27 µg/sq·cm in passive diffusion to 491.37 ± 48.78 µg/sq·cm in sonophoresis group [9]. Tezel and Mitragotri have found that the number of collapse events per unit volume per unit time during the sonophoresis depends on the ultrasound intensity [59]. A 5-min sonophoresis has also been reported to significantly enhance the delivery of calcein across pig skin in vitro [60]. Schoellhammer et al. employed ultrasound operated at 20 kHz and 1 MHz frequency to increase transient cavitation events, create larger localized transport regions in vitro, and significantly enhance transdermal delivery of 4 kDa dextran [54,61]. Low-frequency ultrasound at 20 kHz frequency and 2.4 W/sq·cm intensity was also used to transdermally deliver a significant quantity of antisense oligodeoxynucleotides [45]. Boucaud et al. used 20 kHz ultrasound to drive insulin across hairless rat skin and obtain significant hypoglycemia, which was comparable to subcutaneous injection of 0.5 U insulin [58].

In a report, Le et al. revealed that the application of pulsed-mode ultrasound (20 kHz) through 1% SLS solution to disrupt the skin barrier enhanced the accumulative delivery of heparin by 13-fold [31]. Boucaud and colleagues assessed the effectiveness of LFS (2.5 W/sq·cm and 20 kHz) on the enhanced skin delivery of caffeine and fentanyl. The results showed that the ultrasound treatment significantly enhanced transdermal penetration of fentanyl (about 35-fold greater than passive permeation) and caffeine (about 4-fold greater than passive permeation) across human and hairless rat skin [62]. Mitragotri et al. conducted experiments using LFS (20 kHz, 7 W/sq·cm) to markedly enhance the transport of low-molecular-weight heparin through the skin in vitro (approximately 21-fold greater than the untreated group) [63]. The application of sonophoresis (20 kHz) for skin delivery of insulin has been evaluated by Smith and coworkers. The authors reported a significant increase in the in vitro transport of insulin from Humulin R and Humalog^®^ across human skin, as compared with the nonsonicated group [64]. Interestingly, Mitragotri et al. conveyed that low-frequency ultrasound at 20 kHz was 1000-fold more efficient than sonophoresis operated at high frequencies (1–3 MHz) in enhancing the drug permeation. This greater efficiency of LFS could be attributed to the increased incidence of cavitation events [18,48,65].

#### 3.3.4. The Combination of ITP and Sonophoresis

Interestingly, the combination of anodal ITP and sonophoresis (anodal ITP + LFS, 333.10 ± 37.01 µg/sq·cm) delivered a markedly higher drug amount into the receptor fluid than the use of sonophoresis alone (*n* = 4, *p* < 0.05), while the combination of cathodal ITP and sonophoresis (cathodal ITP + LFS, 178.30 ± 23.79 µg/sq·cm) resulted in a comparable drug delivery as sonophoresis alone (*p* = 0.43). These comparisons appeared consistently in flux and permeability coefficient (Figure 4). Physical treatments led to a notable enhancement of both permeability coefficient and flux than passive permeation (*p* < 0.05) (Table 2). Being employed either alone or in combination with sonophoresis, anodal ITP was always superior to cathodal ITP (*p* < 0.05). The addition of sonophoresis to ITP led to a higher delivery effectiveness than the use of ITP alone.

The combination of ITP and LFS has been investigated to enhance the transdermal delivery of various compounds [31,66,67,68]. In a previous work, we have suggested that ITP could provide a controlled drug-delivery system in which the current was applied to maintain the desired drug level after the ultrasound pretreatment [20]. Le et al. reported a synergistic effect of sonophoresis and ITP on transdermal heparin transport through porcine skin. Specifically, iontophoretic transdermal delivery of heparin across ultrasound-pretreated skin was significantly higher than that observed in ultrasound or ITP alone [31]. Hikima et al. used the combination of sonophoresis (300 kHz, 5.2 W/sq·cm, 5.4% duty cycle) and ITP (0.32 ± 0.03 mA/sq·cm) to treat hairless mouse skin in vitro. The authors suggested that the presence of electrical charge had a notable influence on the permeation of compounds with a molecular weight of less than 500 Da. No significant enhancement was observed with ionized chemicals when sonophoresis was used in tandem with ITP, as compared with ITP alone. The synergistic effect of sonophoresis and ITP was achieved with nonionized chemicals (vitamin B12), chemicals with the increased convective flow, and compounds with molecular weight of more than 1000 Da (irrespective of the electrical charge) [67]. Similarly, Shirouzu et al. reported the combination of ultrasound and ITP synergistically enhanced the permeation flux of chemicals with large molecular weight in vitro (vitamin B12) [66]. This synergy was primarily influenced by electro-osmosis in the stratum corneum layer: the stratum corneum permeability of chemicals increased by LFS and the electro-osmotic water flow by ITP [67]. This observation might explain the superior result of sonophoresis and anodal ITP group (electro-osmosis), as compared with the combination of sonophoresis and cathodal ITP (electrorepulsion). However, the synergistic effects of ultrasound and ITP were absent from chemicals that were delivered solely by electrorepulsion [67]. The ultrasound treatment altered the skin structure and increased the drug diffusivity in the stratum corneum while the ITP produced physical forces of electrorepulsion and electro-osmosis to further the drug permeation. These mechanisms enhanced the movement of not only the therapeutic agents but also water molecules in the stratum corneum. The authors indicated electro-osmosis to be the major factor in the synergistic effects. Furthermore, the synergy was present only in the separate treatment (ITP application on the sonicated skin), not in the simultaneous treatment [67]. Thus, ITP was expected to be applied to the skin samples pretreated with low-frequency ultrasound, as performed in our study. Hikima et al. reported that the penetration-enhancement factor of ionized compounds with anodal ITP was comparable to that with cathodal ITP [67]. The permeation flux of hydrocortisone (nonionized chemical) for the combination of sonophoresis and anodal ITP (1.83 µg/sq·cm/h, 30.5-fold higher than the flux of the passive permeation group) was significantly greater than that of sonophoresis (0.16 µg/sq·cm/h, 2.67-fold) or anodal ITP alone (0.53 µg/sq.cm/h, 8.83-fold) [62,64,66,67]. The combination of sonophoresis and ITP offers significant advantages over the individual treatment, particularly (i) enhancement of transdermal flux, (ii) reduction in the required iontophoretic current to achieve the desired flux, (iii) capacity to transdermally deliver large molecules, and (iv) control of transdermal drug transport.

#### 3.3.5. Steady-State Plasma Concentration of MTX

In this study, we predicted the steady-state plasma level of MTX (*C*_ss_, µg/mL) from the steady-state flux (*J*_ss_, µg/sq·cm/h), the permeation area (*A*, 0.64 sq·cm), and the clearance of MTX from the human body (*Cl*), using Equation (4) [69].

(4) $$C_{ss}=\frac{A\times J_{ss}}{Cl}$$

The use of dermatomed human skin in our study provided meaningful and applicable results to represent human subjects in clinical studies. The clearance of MTX by humans was estimated to be 118 mL/h [70]. We calculated the steady-state plasma levels of MTX and displayed the results in Table 2. Our results were significantly lower than the desired steady-state plasma level (337 ng/mL), which was measured in adult subjects (70 kg) after oral dosing. This result could be explained by the markedly lower topical dose than the oral dose. Furthermore, the combination of anodal ITP and LFS would be preferable to reach the targeted plasma concentration of MTX. The current density, treatment area, and treatment duration of anodal ITP, as well as the drug concentration in the formulation, could be increased to further increase drug delivery whereas the parameters of sonophoresis have been optimized [9]. The enhanced drug permeability should be controlled to avoid possible potential for skin irritation. Even though therapeutic topical dosage of MTX remains unknown, Tiwari and colleagues suggested that 15-min ITP at 0.6 mA/sq·cm current density weekly for four weeks would be efficacious to treat psoriasis [25].

### 3.4. Skin Distribution Studies

**Figure 5:**
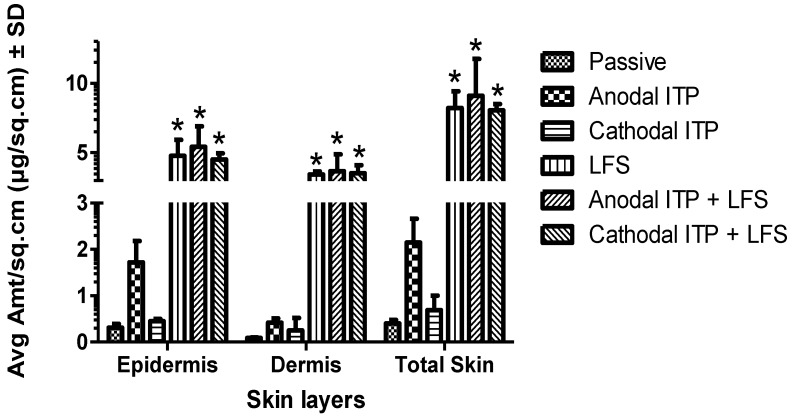
Levels of MTX in dermatomed human cadaver skin (* indicated statistical difference from the passive, anodal, and cathodal ITP, mean ± SD, *n* = 4, *p* < 0.05).

Following the in vitro permeation studies, the distribution of MTX in different layers of human cadaver skin was investigated. We observed that the total amount of MTX in untreated skin (passive, 0.40 ± 0.08 µg/sq.cm) was significantly lower than that in anodal ITP (2.15 ± 0.51 µg/sq.cm, *n* = 4, *p* < 0.05), LFS (LFS, 8.22 ± 1.21 µg/sq.cm, *n* = 4, *p* < 0.05), combination of sonophoresis and anodal ITP (anodal ITP + LFS, 9.10 ± 2.65 µg/sq.cm, *n* = 4, *p* < 0.05), and combination of sonophoresis and cathodal ITP (cathodal ITP + LFS, 8.06 ± 0.45 µg/sq.cm, *n* = 4, *p* < 0.05) (Figure 5). However, cathodal ITP alone delivered a comparable amount of MTX to skin layers (0.69 ± 0.31 µg/sq.cm) with the passive group (*n* = 4, *p* = 0.12). Similarly, no significant difference was reported in the drug level in the skin after the treatment by sonophoresis alone and the combination of cathodal ITP and sonophoresis (*p* = 0.81) (Figure 5). These comparisons were observed consistently in both epidermis and dermis layers of the skin (Figure 5). In another study, Alvarez-Figueroa et al. revealed that the amount of MTX accumulated in the skin increased with increasing drug concentration in the donor solution, increasing current density, or decreasing NaCl concentration in the donor formulation [24]. Levels of ketoprofen in the skin were enhanced from 34.69 ± 7.25 µg in the untreated group to 212.62 ± 45.69 µg in the low-frequency ultrasound group [9]. Tezel et al. employed a 10-min simultaneous application of oligodeoxynucleotides and ultrasound (20 kHz, 2.4 W/sq.cm) to achieve accumulation of 3500 dpm/sq.cm oligodeoxynucleotides in the tissue. Moreover, within 10-min treatment, the authors could deliver 53 µg/sq·cm oligodeoxynucleotides into skin from the donor solution (100 mg/mL oligodeoxynucleotides) [45]. Sonophoresis has been found to increase intradermal accumulation of various molecules in skin layers [9,71]. The relatively high drug levels in skin achieved by our physical enhancement technologies might be sufficient for the treatment of psoriasis. These results suggest that sonophoresis at low frequency (20 kHz) is an effective physical enhancement technique to improve transdermal and intradermal delivery of MTX. Future studies are needed to support this statement since the skin concentration of MTX required for therapeutic effects remains unknown.

## 4. Conclusions

In this study, we employed LFS and ITP to significantly enhance the in vitro skin delivery of MTX transdermally. After these physical treatments, we visualized the skin in dye binding studies. Using in vitro permeation studies on dermatomed human cadaver skin, we reported that the combination of ITP and sonophoresis led to a markedly higher permeation of MTX than the passive group as well as the individual treatment. Thus, LFS and ITP were effective at enhancing the drug delivery into and across human skin in vitro.

## Figures and Tables

**Table 1:** Experimental setup of in vitro permeation studies (*n* = 4).

Compartment	Passive ^a^	Anodal ITP ^b^	Cathodal ITP ^c^	LFS ^d^	Anodal ITP ^b^ + LFS ^d^	Cathodal ITP ^c^ + LFS ^d^
Donor	500 µL MTX 2.0 mg/mL in 10 mM PBS	500 µL MTX 2.0 mg/mL in 10 mM PBS (75 mM NaCl ^f^)	500 µL MTX 2.0 mg/mL in 10 mM PBS	500 µL MTX 2.0 mg/mL in 10 mM PBS	500 µL MTX 2.0 mg/mL in 10 mM PBS (75 mM NaCl ^f^)	500 µL MTX 2.0 mg/mL in 10 mM PBS
Receptor	5 mL PBS (10 mM)	5 mL PBS (10 mM)	5 mL PBS (10 mM, 75 mM NaCl ^f^)	5 mL PBS (10 mM)	5 mL PBS (10 mM)	5 mL PBS (10 mM, 75 mM NaCl ^f^)
Skin pretreatment	NA ^e^	NA ^e^	NA ^e^	Two-min LFS, 100% duty cycle	Two-min LFS, 100% duty cycle	Two-min LFS, 100% duty cycle

^a^ No delivery technology (ITP, sonophoresis), only passive diffusion. ^b^ Anodal ITP, described in Figure 1B. ^c^ Cathodal ITP, described in Figure 1C. ^d^ LFS operating at 20 kHz frequency, two-minute application, 100% duty cycle, 0.3 cm distance between the sonicator probe and the skin surface, 1% (*w*/*v*) sodium lauryl sulfate as the coupling medium, and 30% amplitude, described in Figure 1A. ^e^ No pretreatment on skin tissues. ^f^ NaCl (75 mM) was added to drive the electrochemistry [26].

**Table 2:** Transdermal delivery, lag time, flux, permeability coefficient, and steady-state plasma concentration of in vitro permeation of MTX though dermatomed human cadaver skin (mean ± SD, *n* = 4).

Group	*Q*_24_^a^ (µg/sq·cm)	Lag Time ^b^ (h)	*J*_ss_ (µg/sq·cm/h) ^c^	*K*_p_ (cm/h) ^d^ × 10^−4^	*C*_ss_ (ng/mL) ^e^
Passive	0.00 ± 0.00	0.00 ± 0.00	0.00 ± 0.00	0.00 ± 0.00	0.00 ± 0.00
Anodal ITP	4.74 ± 0.62	3.57 ± 1.07	0.05 ± 0.03	0.26 ± 0.14	0.28 ± 0.15
Cathodal ITP	0.54 ± 0.07	0.20 ± 0.05	0.01 ± 0.00	0.07 ± 0.02	0.08 ± 0.02
LFS	161.92 ± 30.06	−5.09 ± 1.03	6.81 ± 1.31	34.04 ± 6.55	36.92 ± 7.11
Anodal ITP + LFS	333.10 ± 37.01	11.83 ± 10.77	13.42 ± 1.95	67.08 ± 9.73	72.77 ± 10.55
Cathodal ITP + LFS	178.30 ± 23.79	−15.93 ± 16.74	8.03 ± 1.17	40.14 ± 5.85	43.54 ± 6.35

^a^ Cumulative amount of MTX (*Q*_24_) permeated through a unit of diffusion area in 24 h. ^b^ Lag time—calculated as the x-intercept of the linear portion of the permeation curve (*R^2^* > 0.90). ^c^ Steady-state flux (*J*)—calculated from the linear slope of the permeation curve. ^d^ Permeability coefficient (*K*_p_)—calculated using an equation: *K*_p_ = *J*/*CA*, where *K_p_* is the permeability coefficient (cm/h), *J* is the steady-state flux (µg/h), *C* is the MTX concentration in the donor (µg/mL), and *A* is the permeation area (0.64 sq.cm). ^e^ Steady-state plasma concentration (*C_ss_*)—calculated using an equation: *C*_ss_ = (*A* × *J*_ss_)/*Cl*, where *C*_ss_ is the steady-state plasma concentration (µg/mL), *A* is the permeation area of skin (0.64 sq·cm), *J*_ss_ is the steady-state flux (µg/sq·cm/h), and *Cl* is the clearance of MTX from the body.

## Abbreviations

DI water	Deionized water
ITP	Iontophoresis
LFS	Low-frequency sonophoresis
MTX	Methotrexate
OCT	Optimal cutting temperature
PBS	Phosphate Buffer Saline
RP-HPLC	Reversed-Phase High-Performance Liquid Chromatography
SD	Standard Deviation
SLS	Sodium Lauryl Sulfate
TEWL	Transepidermal Water Loss
